# Transcriptome profiling reveals novel gene expression signatures and regulating transcription factors of TGF *β*‐induced epithelial‐to‐mesenchymal transition

**DOI:** 10.1002/cam4.719

**Published:** 2016-06-18

**Authors:** Liutao Du, Shota Yamamoto, Barry L. Burnette, Danshang Huang, Kun Gao, Neema Jamshidi, Michael D. Kuo

**Affiliations:** ^1^ Department of RadiologyThe David Geffen School of Medicine at UCLALos AngelesCalifornia90095; ^2^ Department of NeurologyUCLALos AngelesCalifornia90095; ^3^ Department of BioengineeringUniversity of California‐Los AngelesLos AngelesCalifornia90095

**Keywords:** Breast, epithelial to mesenchymal transition, genomics, transcription factor, non‐small‐cell lung cancer

## Abstract

Dysregulated epithelial to mesenchymal transition (EMT) in cancer cells endows invasive and metastatic properties upon cancer cells that favor successful colonization of distal target organs and therefore play a critical role in transforming early‐stage carcinomas into invasive malignancies. EMT has also been associated with tumor recurrence and drug resistance and cancer stem cell initiation. Therefore, better understanding of the mechanisms behind EMT could ultimately contribute to the development of novel prognostic approaches and individualized therapies that specifically target EMT processes. As an effort to characterize the central transcriptome changes during EMT, we have developed a Transforming growth factor (TGF)‐beta‐based *in vitro* EMT model and used it to profile EMT‐related gene transcriptional changes in two different cell lines, a non‐small cell lung cancer cell line H358, and a breast cell line MCF10a. After 7 days of TGF‐beta/Oncostatin M (OSM) treatment, changes in cell morphology to a mesenchymal phenotype were observed as well as concordant EMT‐associated changes in mRNA and protein expression. Further, increased motility was noted and flow cytometry confirmed enrichment in cancer stem cell‐like populations. Microarray‐based differential expression analysis identified an EMT‐associated gene expression signature which was confirmed by RT‐qPCR and which significantly overlapped with a previously published EMT core signature. Finally, two novel EMT‐regulating transcription factors, IRF5 and LMCD1, were identified and independently validated.

## Introduction

The connection between epithelial mesenchymal transition (EMT) and cancer progression has been implicated in several types of cancer, including breast, prostate, pancreatic, and liver cancers 1, 2, 3. Indeed, mounting evidence suggests that co‐opting of EMT, an evolutionarily conserved process required for tissue morphogenesis during embryonic development, plays a critical role in tumor invasion, acquisition of cancer stem cell‐like properties, and the development of metastasis and drug resistance 4. While loss of E‐cadherin expression represents a primal event in EMT, we increasingly appreciate that EMT is a complex process that is orchestrated by activation and repression of a growing and still incomplete list of genes, proteins, and transcriptional factors including ZEB1, SNAIL, TWIST, CDH2, and VIM among others 5, 6, 7. Although several different EMT‐related gene expression signatures (GES) have been reported to date attempting to capture key EMT‐associated genes and regulators of EMT, they have been limited in part by variations observed between cell lines, limited concordance between different EMT induction models, and lack of functional validation 8, 9, 10, 11, 12. Therefore, additional studies are still needed to identify both a robust, common set of EMT‐associated genes and regulators in order to help improve our understanding of EMT, which could serve to drive future prognostic approaches and individualized therapies.

Here, we have developed a non‐cell autonomous TGF‐beta‐based EMT induction model to profile and characterize the transcriptomic changes involved in the EMT process. After validation of our EMT induction model, we performed gene expression microarray analysis and identified a common set of EMT‐related genes in two different cell lines which shows strong concordance with a previously reported EMT‐GES. Finally, we report on two new EMT‐regulating transcription factors (TFs) that have potential direct regulation of this unique process.

## Materials and Methods

### Cell culture and EMT induction

H358, MCF10a, and A549 cells used in this study were purchased from the American Type Culture Collection (ATCC) and grown as per ATCC protocols and cultured in an incubator at 37°C in the presence 5% CO_2_. For the EMT induction, untreated cells (preEMT) were treated with a combination of TGF‐beta (10 ng/mL) and OSM (50 ng/mL) for 7 days (postEMT) and then subsequently analyzed for EMT induction. Cell medium was changed every 3 days and fresh TGF‐beta and OSM were added with each medium change. TGF‐beta and OSM were purchased from Cell Signaling Technology (Danvers, MA).

### Microarray and IPA pathway analysis

All RNA samples for expression analysis were isolated using RNAeasy kit (Qiagen, Valencia, CA) and inspected for quality using an Agilent 2100 Bioanalyzer (Agilent, Santa Clara, CA) and gel electrophoresis in the University of California Los Angeles (UCLA) Clinical Microarray Core (UCMC). All the RNAs were confirmed to be high quality with RIN scores over 8. Gene expression profiling was performed using Agilent 8 × 60 k arrays (Agilent, Santa Clara, CA). The microarray data was processed with the edgeR package to analyze differential expression of genes 13. Ingenuity pathway analysis (IPA) software (Qiagen, Redwood City, CA) was used for analysis of the signaling and metabolic pathways, molecular networks, and biological processes most significantly perturbed in the gene expression datasets 14. IPA was also used to predict TFs, which could be responsible for gene expression changes and whether those TFs are activated or inhibited in EMT.

### RT‐qPCR and EMT‐gene‐qPCR array

Total RNA was extracted using RNAeasy kit (Qiagen, Valencia, CA). Reverse‐transcription reactions were catalyzed by Superscript III reverse transcriptase (Invitrogen, Carlsbad, CA). Subsequently, qPCR was performed using SensiFAST SYBR Lo‐ROX Kit (Bioland Scientific, Paramount, CA) on an ABI Fast 7500 Real‐Time PCR System (Life Technologies, Grand Island, NY). All the qPCR primers were designed and validated by standard curve approaches. Relative quantification values were calculated using the ddCt method after confirmation of equivalent amplification efficiencies between target genes and the reference gene. The EMT‐gene qPCR array was purchased from Invitrogen (Life Technologies, Grand Island, NY). For all qPCR, two‐step PCR cycling condition was used: 95°C for 2 min followed by 40 cycles of 95°C for 5 sec, 60°C for 20 sec. Primer sequences are available upon request.

### siRNA knockdown and western blots

Cells were treated with siRNA against each individual gene at concentrations described per Lipofectamine^®^ RNAiMAX protocol (Invitrogen, Carlsbad, CA). The sequences of the two IRF5 siRNAs used were previously published (Catalog #16708; Ambion, Austin, TX) and were purchased from Invitrogen (Life Technologies, Grand Island, NY). The predesigned triple siRNAs to LMCD1 were ordered from Integrated DNA Technologies Inc (Coralville, Iowa) and #1 and #2 were validated and used in experiments. For western blotting, cells were collected and whole cell lysate was prepared with RIPA buffer and used for target protein analysis. All antibodies were purchased from Cell Signaling (Danvers, MA) and were used for overnight incubation at 4°C according to manufacturers' instructions.

### Flow cytometry analysis

Cells were suspended in phosphate‐buffered saline (PBS) with 20% fetal bovine serum (FBS) and incubated with relevant antibodies for 1 h on ice. Cells were then washed and resuspended with PBS + FBS for flow cytometry analysis (FACS) analysis on FACScanX (BD Biosciences, San Jose, CA) at the UCLA Flow Cytometry Core Laboratory. Antibodies against CD44 (PE‐conjugated) and CD24 (APC‐conjugated) were purchased from BD Biosciences (San Jose, CA); antibody against CD133 (PE‐conjugated) was purchased from Miltenyi Biotec, (Cologne, Germany); antibody against E‐cadherin (APC‐conjugated) was purchased from Biolegend (San Diego, CA).

### Cell invasion assay

Cell invasion was measured by CytoSelect^™^ 24‐Well Cell Migration and Invasion Assay (Cell Biolabs, San Diego, CA) according to the manufacturer's instructions. Briefly, the basement membrane layer of the cell culture inserts was rehydrated with serum‐free media for 1 h. Media (10% FBS, 500 *μ*L) was added to the lower well of the migration plate. Cells were prepared and suspended in serum‐free media and were added to the inside of each insert and incubated for 48 h in an incubator. The media was aspirated from the inside of the insert, and nonmigratory cells were then removed. The insert was transferred to a clean well containing 400 *μ*L of cell stain solution and incubated for 10 min and washed. Each insert was then transferred to an empty well and extraction solution was added at 200 *μ*L per well, and then incubated for 10 min in an orbital shaker. From each sample, 100 *μ*L was transferred to a 96‐well microtiter plate and measured for OD_560 nm_ (BioTek, Winooski, VT).

## Results

### Identification of an EMT‐related gene expression signature by microarray analysis

**Figure 1:**
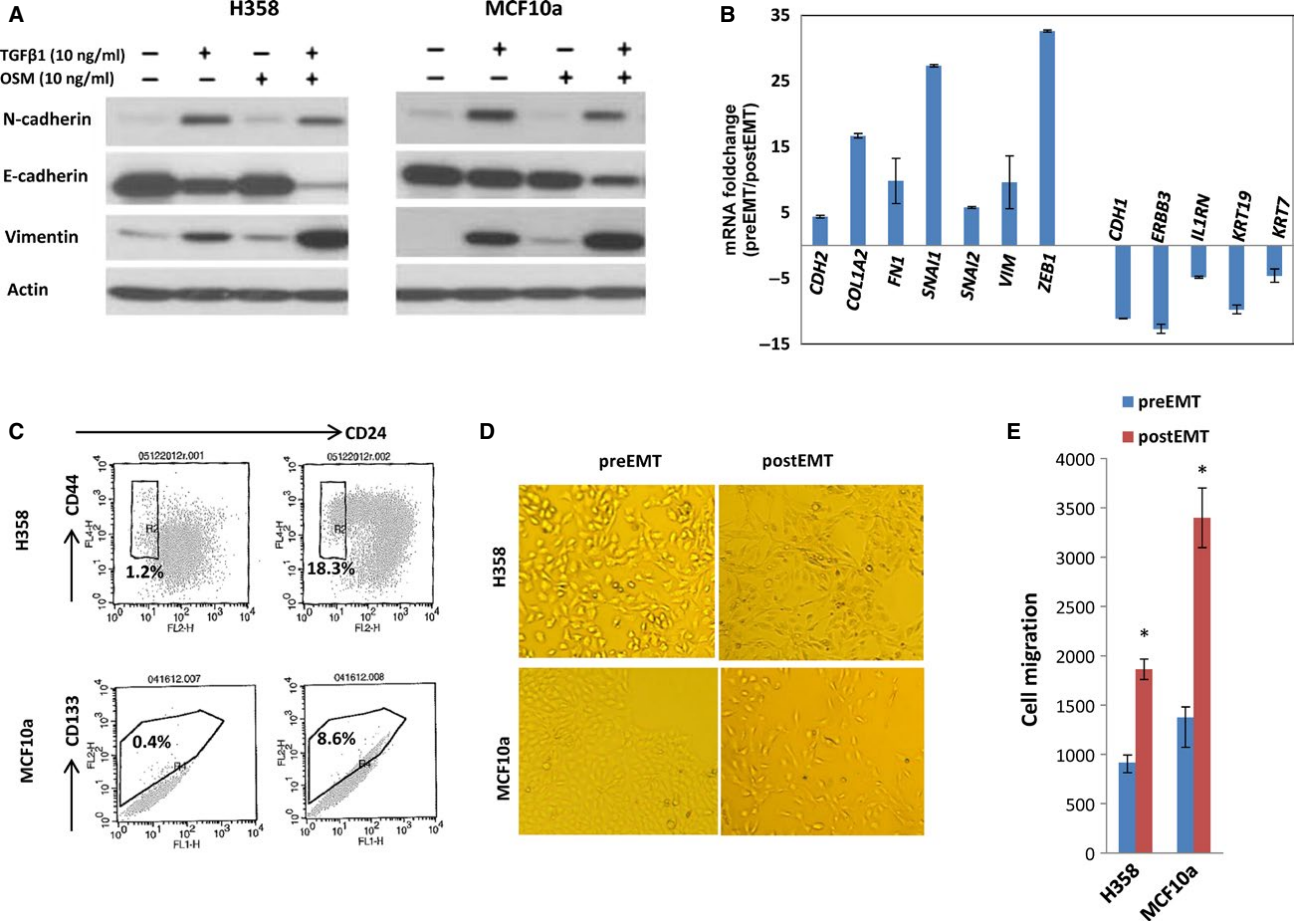
An optimized TGF‐beta/OSM approach to efficiently induce epithelial mesenchymal transition (EMT) in H358 and MCF10a cells. (A) Western Blot of EMT protein for H358 cells (left) and for MCF10a cells (right). After 7 days of exposure to TGF‐beta/OSM, a clear loss of epithelial biomarker E‐cadherin and increased expression of mesenchymal proteins vimentin and N‐cadherin are shown; (B) RT‐qPCR quantification of 12 important EMT genes in H358 cells treated with TGF‐beta/OSM; (C) TGF‐beta/OSM‐induced CSC‐like cells: CD44^+^/CD24^−^ subpopulation in H358 cells and CD133^+^ subpopulation in MCF10a cells. (D) Mesenchymal morphological changes; (E) cell invasiveness. *P < 0.05.

We optimized a TGF‐beta‐based EMT induction approach for use as an efficient and reproducible means of inducing EMT in different cell lines (H358 and MCF10a). A 7‐day treatment of TGF‐beta/OSM improved the efficiency of EMT induction, as compared to a single TGF‐beta or OSM treatment, as demonstrated by protein expression changes of important EMT markers including E‐cadherin, Vimentin, and Fibronectin (Fig. 1A; left panel: H358; right panel: MCF10a). TGF‐beta/OSM was also more efficient for inducing EMT than other known EMT‐inducing cytokines, such as HGF, FGF, and IGF (data not shown). In the H358 cells treated with TGF‐beta/OSM, expression of important EMT genes, such as *CDH2*,* COL1A2*,* FN1*,* SNAI1*,* SNAI2*,* VIM*,* CDH1*, and *ERBB3,* were all changed as expected after EMT induction (Fig. 1B). TGF‐beta/OSM also significantly increased the CSC‐like subpopulation cells in both cell lines (CD44^+^/CD24^−^ or CD44^+^ in H358; CD133^+^ in MCF10a, Fig. 1C), induced mesenchymal morphological changes (Fig. 1D) and promoted cell invasiveness (Fig. 1E), which are consistent with previous reports.

To evaluate the transcriptomic changes during EMT in H358 and MCF10a cells, we used three independent sets of preEMT and postEMT RNA as biological triplicates for each cell line which were used for microarray‐based gene expression profiling. We identified >2000 genes associated with EMT (fold change >2, and *P* < 0.05). The top 50 significantly differentially expressed genes were listed in Table S1, all with fold change >16. A common GES of differentially expressed genes shared between the two cell lines consisting of 571 genes, with 269 genes upregulated and 302 genes downregulated (fold change >2, and *P* < 0.05) was identified (Table S2). Among these genes, ~127 genes had fold change >4 in both cell lines. The top‐10 upregulated common genes are *SERPINB3, SERPINB4, SCG5, FAP, MMP9, GPR68, SERPINE1,CXCR7, ADAM19*,* and SLAMF8*; the top‐10 downregulated common genes are *KRT15 PSCA, FGFBP1, FXYD3, MAL2, GPR110, C10orf116, S100P, SUSD2*,* and MYO5C*. After comparing our 571‐EMT‐GES with a previously published 246‐gene EMT‐core‐GES in breast cancer cells, we found 58 overlapping genes (=5 × 10^−42^, hypergeometric distribution) (Table S3), suggesting that these genes are a part of a core set of EMT‐genes that are neither tissue or cell type specific 8.

### Targeted validation of EMT transcriptomic profiles with RT‐qPCR

**Figure 2:**
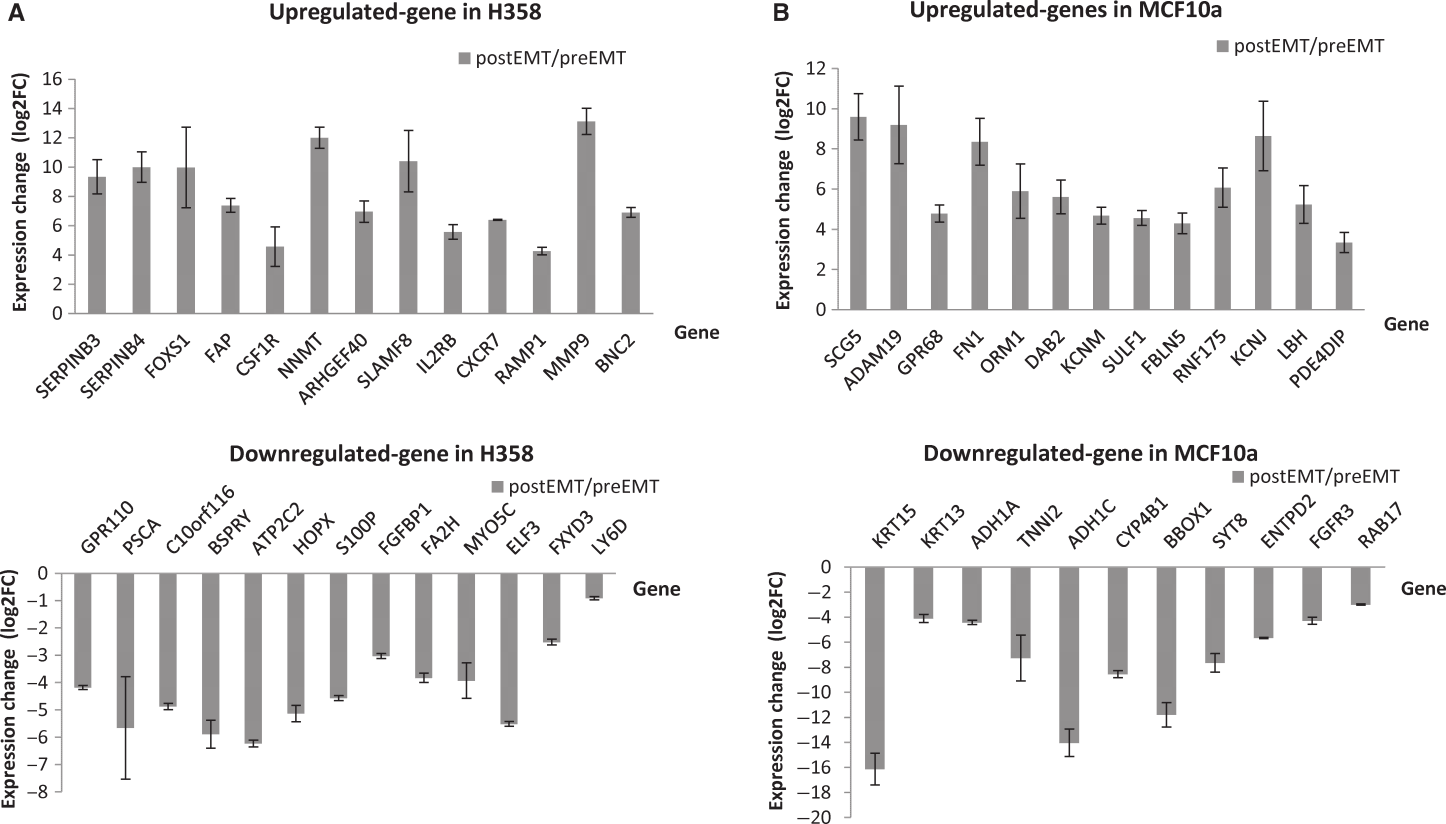
RT‐qPCR confirmation of the differential expression of selected top genes in H358 and MCF10a cells. Cells were induced with TGF‐beta/OSM for 7 days and RNAs were prepared. The top genes differentially expressed were selected in each cell line and validated by RT‐qPCR to confirm their expression differences (GADPH was used as reference control). (A) Expression confirmation of selected genes in H358 cells; (B) Expression confirmation of selected genes in MCF10a cells. All tested genes were significantly differentially expressed after epithelial mesenchymal transition‐induction (*P* < 0.05).

To confirm the accuracy of our EMT gene expression signature, we selected 25 genes from the top 50 upregulated and downregulated genes from our EMT GES and conducted RT‐qPCR using newly prepared samples. The differential expression of all of these selected genes was successfully confirmed by RT‐qPCR in this independent data set (Fig. 2). To further validate our microarray data results, we evaluated the mRNA levels of 65 genes that were previously reported to be related to EMT using a commercial RT‐qPCR array and compared with our microarray data results. The expression changes for 62 out of 65 genes were validated (Table S4). The three genes that were not confirmed by the RT‐qPCR EMT array had relatively low expression changes close to the cut‐off ratio which was set at a fold change of 2. Together, these results confirm the reproducibility of our EMT‐GES in independent samples.

### EMT‐related TFs and signaling networks identified in the EMT‐GES

From our 571‐EMT‐GES, we found 34 TFs significantly differentially expressed during EMT induction (Table 1). The important known EMT‐activating TFs, such as ZEB1, ZEB2, SNAIL1, SNAIL2, RUNX2, and ETS1, were all found upregulated, while known negative TF regulators of EMT, such as SMAD3, MITF, and ETF, were all downregulated in postEMT cells in concordance with the literature 16, 17. IPA also identified a number of TFs that did not show significant differential expression changes in postEMT cells but were predicted to be activated or inhibited based on the expression profiles of their target genes. For example, *TWIST1, TWIST2, HDAC6, MUC1, ETS2, NFkB, Jun, SP1*, and *AP1* were previously reported as enhancers of EMT, and were found activated in postEMT cells. Other TFs, such as *ER, SPDEF*, and *HMGA1*, were previously reported as repressors of EMT, and were found repressed in this study 18, 19, 20. Interestingly, SMAD7 was previously reported as an EMT repressor; we found that *SMAD7* gene expression was consistently elevated after EMT induction and positively correlated with postEMT 21. IPA analysis also revealed that more than 10 networks (Nt) were activated during EMT; the top five common networks were shown in Fig. S1.

**Table 1:** TFs that expressed differently in both H358 and MCF10a cells

Gene	Genbank ID	Log2FC in H358	*P*‐value in H358	Log2FC in MCF10a	*P*‐value in MCF10a	Averaged log2FC
ZEB1	NM_001128128	3.42	7.67E‐06	1.64	1.22E‐02	2.53
SNAI1	NM_005985	3.17	2.55E‐05	4.1	7.50E‐05	3.64
SNAI2	NM_003068	2.6	7.61E‐03	2.21	1.21E‐02	2.41
MAF	NM_001031804	2.48	6.02E‐04	1.63	6.16E‐04	2.06
ZEB2	NM_014795	2.42	5.76E‐04	1.3	7.03E‐03	1.86
ELK3	NM_005230	1.92	1.25E‐03	1.25	1.50E‐03	1.59
E2F7	NM_203394	1.9	1.54E‐02	1.04	6.46E‐03	1.47
SMAD7	NM_005904	1.86	3.15E‐03	2.47	1.02E‐03	2.17
ETS1	NM_005238	1.85	1.49E‐02	1.42	1.97E‐03	1.63
SMARCA1	NM_003069	1.77	1.29E‐03	1.2	2.00E‐02	1.49
RAI14	NM_015577	1.71	6.66E‐03	1.74	1.93E‐04	1.72
TGFB1I1	NM_001042454	1.63	1.88E‐03	2.12	2.03E‐03	1.88
**LMCD1**	NM_014583	1.58	1.34E‐02	4.5	8.46E‐05	3.04
SKIL	NM_005414	1.56	1.20E‐04	2.34	1.66E‐03	1.95
JARID2	NM_004973	1.44	5.30E‐03	1.34	1.32E‐02	1.39
RUNX2	NM_004348	1.3	2.35E‐02	3.57	7.23E‐04	2.44
JAZF1	NM_175061	1.23	7.60E‐03	1.24	1.57E‐03	1.23
ID2	NM_002166	1.22	3.97E‐02	1.5	4.24E‐02	1.36
RNF2	NM_007212	1.11	5.58E‐03	1.31	1.58E‐02	1.21
MITF	NM_198159	−1	4.39E‐02	−1.04	2.29E‐02	−1.02
NFIB	NM_005596	−1	1.74E‐02	−2.88	2.18E‐04	−1.94
ZNF488	NM_153034	−1.02	8.26E‐03	−1.19	3.94E‐02	−1.11
EIF1AX	NM_001412	−1.03	1.54E‐02	−1.05	3.77E‐03	−1.04
PIR	NM_003662	−1.19	3.37E‐02	−1.39	3.72E‐03	−1.29
EEF1A2	NM_001958	−1.39	1.06E‐02	−1.03	2.99E‐02	−1.21
TBL1X	NM_005647	−1.4	2.93E‐03	−1.25	7.93E‐03	−1.32
ELL3	NM_025165	−1.42	1.94E‐02	−2.41	8.62E‐05	−1.92
IKZF2	NM_001079526	−1.86	1.28E‐03	−1.48	8.96E‐03	−1.67
TOB1	NM_005749	−1.88	1.76E‐03	−1.72	6.52E‐03	−1.8
LSR	NM_205834	−1.93	1.94E‐04	−1.76	7.17E‐03	−1.85
SMAD3	NM_005902	−1.94	1.06E‐03	−1.27	3.96E‐03	−1.61
**IRF5**	NM_001098627	−2.37	7.92E‐04	−1.33	9.89E‐04	−1.85
E2F2	NM_004091	−2.71	1.80E‐02	−2.62	9.89E‐05	−2.66
OVOL2	NM_021220	−3.53	2.66E‐05	−1.27	8.67E‐03	−2.4
EHF	NM_012153	−4.26	2.49E‐06	−1.85	8.14E‐03	−3.05

### Novel EMT‐regulating TFs identified and validated by functional studies

**Figure 3:**
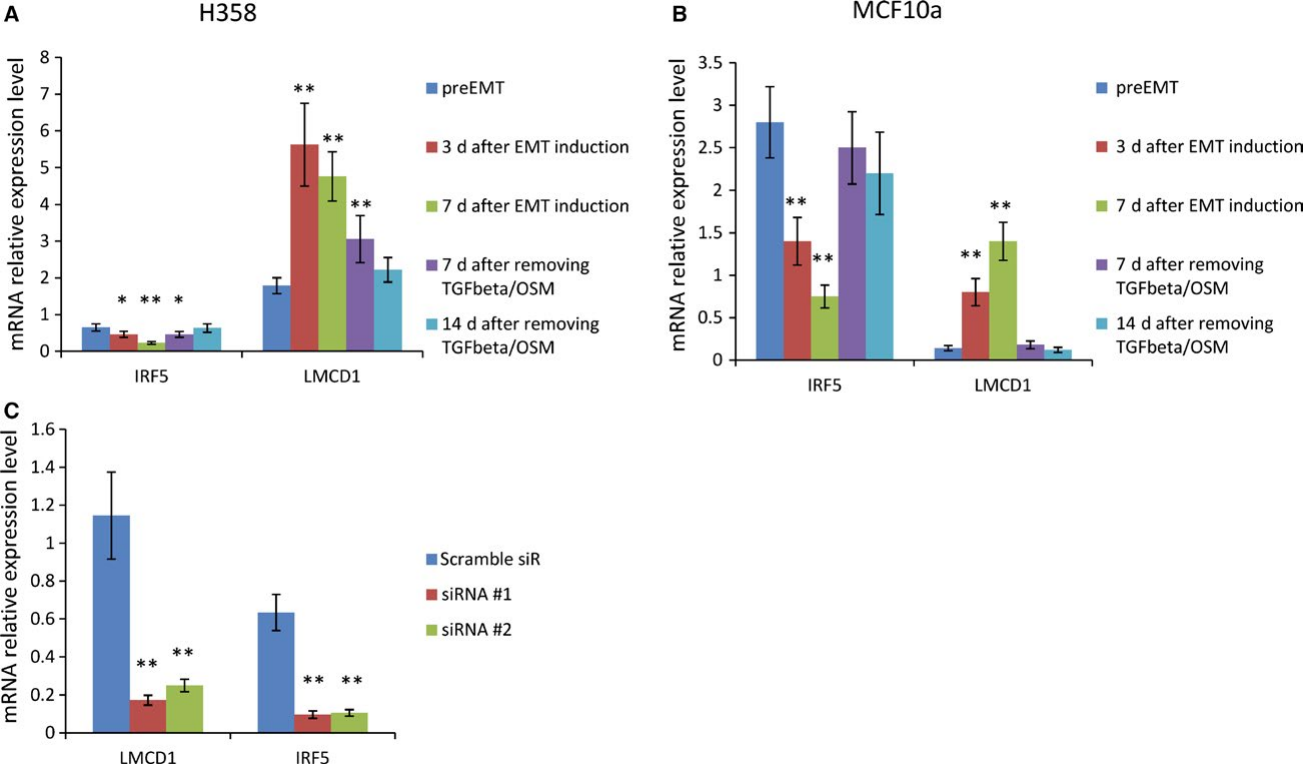
Time course of gene expression patterns of IRF5 and LMCD1 after 7 days of mesenchymal transition (EMT) induction with TGF‐beta/OSM followed by removal of TGF‐beta/OSM for 14 days and validation of siRNA‐mediated knockdown efficiency of IRF5 and LMCD1 in H358 and MCF10a cells. (A) The relative expression levels of *IRF5* and *LMCD1* were measured by RT‐qPCR in H358 and (B) MCF10a cells over a 21 days EMT induction‐reversion time course in which the preEMT cells were first induced to undergo EMT via TGF‐beta/OSM‐based induction followed by removal of TGF‐beta/OSM at day 7 (*P < 0.05; **P < 0.001, as compared to preEMT cells). (C) Confirmation of knockdown efficiency of selected siRNAs on *LMCD* and *IRF5* gene expression in H358 cells; two siRNAs were optimized and used to knockdown their relative genes at 4 nmol/L (**P < 0.001, as compared to scramble‐siR control).

**Figure 4:**
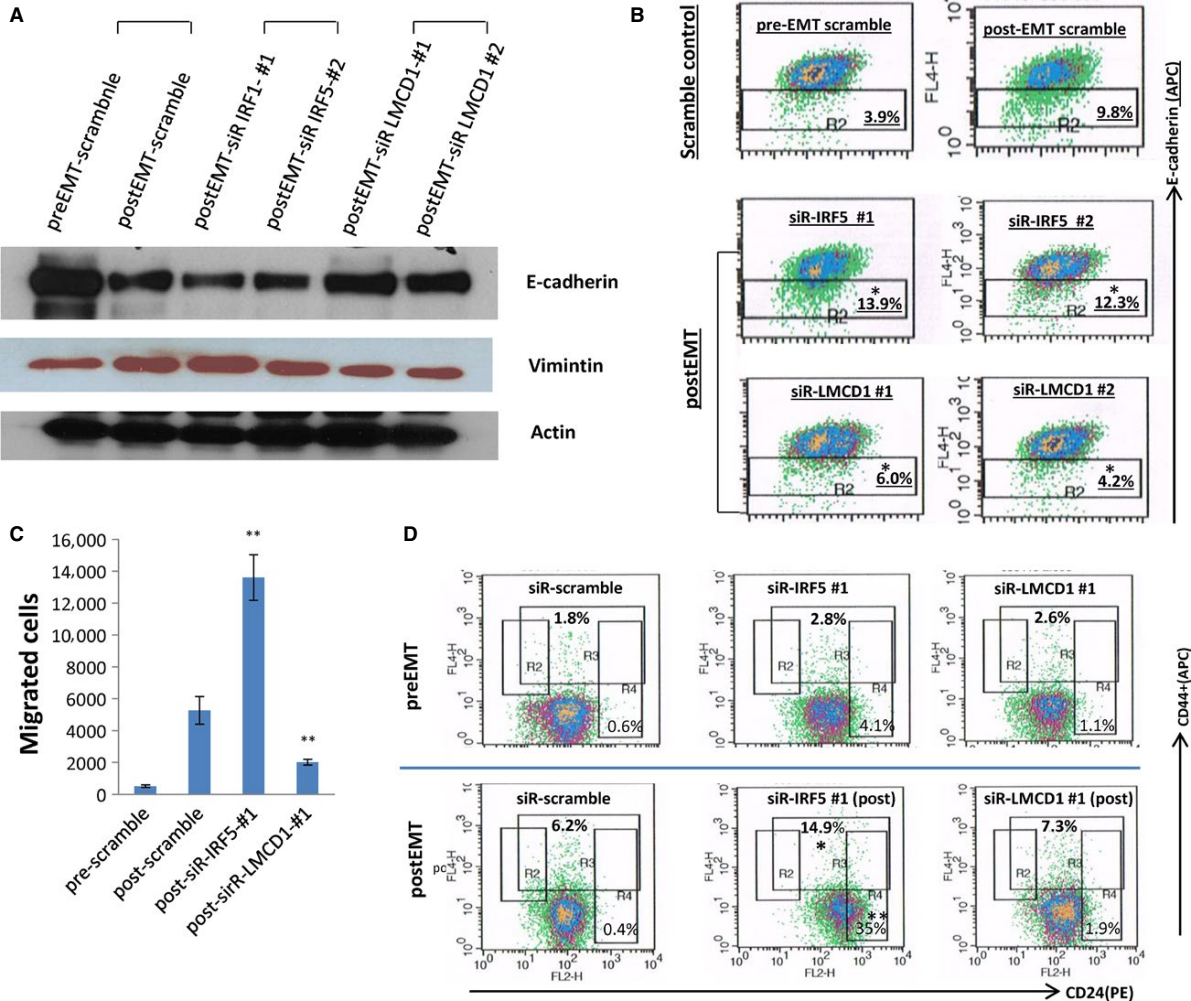
Effects of siRNA‐mediated knockdown of IRF5 and LMCD1 on TGF‐beta/OSM‐induced epithelial mesenchymal transition (EMT) in H358 cells. H358 cells were treated with each indicated siRNA (6 nmol/L) for 48 h and followed by induction of EMT using TGF‐beta/OSM for 3 days. Cells were then collected for follow‐up analysis. (A) Effects of siRNAs on EMT protein markers; (B) Effects of siRNAs on E‐cadherin low‐expressing subpopulation (labeled with anti‐E‐cadherin‐APC antibody); (C) Effects of siRNAs on TGF‐beta/OSM‐mediated invasiveness; (D) Effects of siRNAs on CD24^−^ and CD44^+^ subpopulations (labeled with CD24‐PE and CD44‐APC antibodies). Note: *P < 0.05; **P < 0.001, as compared to the postEMT scramble‐siR control.

As shown in Table 1, certain TFs were found to be differentially expressed after EMT induction, however, their roles in EMT have not been previously reported. We focused on two TFs that may be involved in EMT regulation: IRF5 and LMCD1. *IRF5*′s expression was significantly inhibited after EMT, whereas *LMCD*1*′s* was significantly enhanced (Table 1). We first confirmed that *IRF5* gene expression levels started to gradually decrease after EMT was induced by TGF‐beta/OSM treatment, and then began to revert upon removal of the TGF‐beta/OSM stimulus. Conversely, *LMCD1* gene expression levels increased after EMT was induced, and then decreased after the EMT process was reversed (Figs. 3A and B). To study their potential roles in EMT regulation, we used gene‐specific siRNAs to knockdown each of these individual TFs in H358 cells, and then exposed the siRNA‐treated cells to TGF‐beta/OSM to examine if the TGF‐beta‐mediated EMT was subsequently hindered or enhanced. To validate the siRNA knockdown, two different siRNAs were optimized and knockdown efficiency for each siRNA confirmed by RT‐qPCR (Fig. 3C). We found that inhibition of *IRF5*‐enhanced TGF‐beta mediated EMT progression, while conversely, knockdown of *LMCD1* repressed TGF‐beta‐mediated EMT induction, as evidenced by the expression pattern of EMT protein biomarkers (Fig. 4A). FACS analysis of cell surface E‐cadherin also indicated that knockdown of *LMCD1* significantly reduced the E‐cadherin‐low‐expressing cell subpopulation (from 9.8% to 4.2% (siR#1) or to 6% (siR#2), *P* < 0.05), while knockdown of IRF5 increased the E‐cadherin‐low‐expressing cell subpopulation (from 9.8% to 13.9% (siR#1), or to 12.3% (siR#2), *P* < 0.05) (Fig. 4B). Knockdown of *LMCD1* also significantly reduced cell invasiveness of TGF‐beta/OSM‐treated cells (*P* < 0.05) (Fig. 4C), but no significant effect was seen on the CD24^+^ or CD44^+^ cell subpopulations (Fig. 4D), as compared to the scrambled control‐siRNA‐treated cells. Conversely, knockdown of IFR5 significantly promoted cell invasiveness of TGF‐beta/OSM‐treated cells (Fig. 4C), and dramatically increased the CD24^+^ subpopulation (from 0.4% to 35%, *P* < 0.001) and also increased the CD44^+^ subpopulation (from 6.2% to 14.9%, *P* < 0.05) (Fig. 4D), indicating its role in negative regulation of both CD24‐ and CD44‐expressing cell populations during EMT. Further, cells treated with siR‐IRF5 also showed a more EMT mesenchymal pattern compared with control cells after EMT induction, while cells treated with siR‐LMCD1 had slightly reduced, but recognizable EMT morphologic features (Fig. S2, in both H358 and MCF10a).

**Figure 5:**
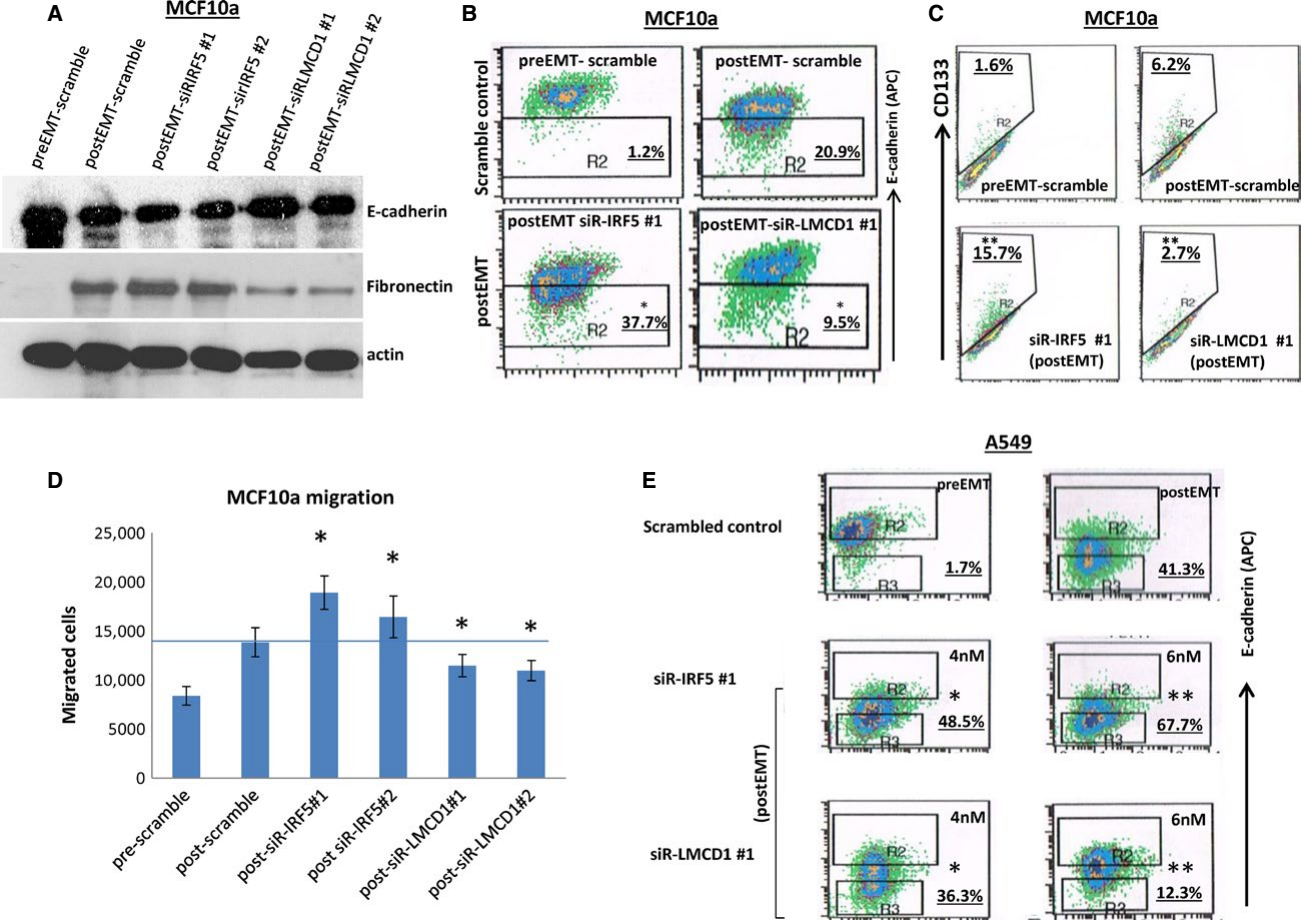
Effects of siRNA‐mediated knockdown of IRF5 and LMCD1 on TGF‐beta/OSM‐induced epithelial mesenchymal transition (EMT) in MCF10a and A549 cells. Cells were treated with each siRNA for 48 h at indicated concentrations followed by induction of EMT using TGF‐beta/OSM for 3 days. Cells were then collected for follow‐up analysis. (A) Effects on EMT protein markers in MCF10a cells (4 nmol/L of siRNA); (B) Effects on E‐cadherin‐low‐expressing subpopulation in MCF10a cells, (4 nmol/L of siRNA); (C) Effects of siRNAs on CD133^+^ cell subpopulations (4 nmol/L of siRNA); (D) effects on invasion; and (E) Effects on E‐cadherin‐low‐expressing subpopulation in A549 cells (4 nmol/L and 6 nmol/L for each siRNA). Note: *P < 0.05; **P < 0.001, as compared to the postEMT scramble‐siR control.

To investigate whether these effects observed in H358 cells were cell type‐dependent or not, we additionally conducted siRNA knockdown in breast MCF10a cells and a second lung cancer line, A549, that were both induced to undergo EMT by the same TGF‐beta/OSM approach. Again, similar effects were observed in both cell lines. We also observed similar findings between MCF10a and H358 cells after knockdown of these two TFs; knockdown of IRF5 enhanced the EMT processes, while knockdown of LMCD1 inhibited EMT. These are indicated by the changes of critical EMT protein expression patterns (Fig. 5A), the E‐cadherin‐low‐expressing cell subpopulation (Fig. 5B), CD133^+^ subpopulation (Fig. 5C), and changes in invasion (Fig. 5D). Similar effects were also observed on E‐cadherin‐low cell population in A549 cells (Fig. 5E). Taken together, these data suggested that in a TGF‐beta‐mediated model of EMT, IRF5 may act as an important EMT repressor while LMCD1 may act as an EMT enhancer. IRF5 may also play important roles in negative regulation of CD24 and CD44 subpopulations during EMT.

## Discussion

EMT is believed to play a critical role in the cellular transformation to an invasive and ultimately metastatic cancer phenotype. However, a detailed understanding of the genes involved and their regulation is incompletely understood. Herein, we present an efficient model of EMT induction with TGF‐beta/OSM in MCF10a and H358 cell lines. We show that this model faithfully recapitulates characteristics associated with EMT including appropriate changes in mRNA and protein expression of key EMT‐associated markers as confirmed by both PCR and western analysis, changes in cell morphology, changes in distribution of CSC‐cell populations, and alterations in cell motility. Using this model, we then characterized gene expression patterns involved with EMT transduction in both a cancerous and noncancerous cell line from two different tissue sites and define a shared EMT‐associated GES from which we then independently validated the top differentially expressed genes by RT‐qPCR. The strong overlap between the 58 genes identified from our EMT GES with a published core EMT GES suggest that these 58 genes may be part of a larger core set of EMT‐genes that are neither tissue or cell type specific 8. Further studies will be required to confirm this, but these results are nonetheless intriguing.

Interestingly, we found more than 10 networks activated during EMT. Nt 1 was centered on TGF‐beta and involves nuclear proteins such as nup210, lipolysis‐stimulated lipoprotein receptor (LSR), V‐Maf avian musculoaponeurotic fibrosarcoma oncogene homolog (MAF) and FILIp1L (Fig. S1). These proteins have been related to EMT regulation and cellular differentiation 22, 23, 24, 25. Nt2 was centered on NFkB and involved genes such as NFkB early‐response gene IER3, secreted signaling molecules bone morphogenetic protein (BMP), and TF ETS homologous factor (EHT) that belongs to an ETS TF subfamily characterized by epithelial‐specific expression and which may be involved in epithelial differentiation and carcinogenesis 26, 27, 28, 29. Nt3 was closely related to cancer and cellular movement being involved with the WNT pathway and ‘cadherin switch’ (repression of E‐cadherin and expression of N‐cadherin). Nt4 is related to cellular assembly and organization with vimentin and cytokeratin, and Nt5 involved signaling of cellular movement, cell‐to‐cell signaling, and interaction pathways regulated by SNAI1, SNAI2 and ZEB1, TGF‐beta, WNT and BMP signaling pathways, which are believed to regulate embryonic stem cell progression. These data further substantiate the potential role these genes may have in EMT and CSC regulation 30.

Most interestingly, we identified two new TFs, IRF5 and LMCD1 that may play an important role in EMT induction. Our data suggest that LMCD1 acts as an enhancer of TGF‐beta‐dependent EMT processes, while IRF5 functions as a negative regulator of EMT. LMCD1 is a member of the LIM protein family containing two C‐terminal LIM domains, a central PET domain, and an N‐terminal cysteine‐rich region 31. LMCD1 was previously described as a transcriptional co‐repressor of GATA6, whereas its role in cytoskeletal compartments remains to be elucidated 32. LMCD1 E135K somatic mutation was associated with cell migration and tumor metastasis in hepatocellular carcinoma (HCC); its up‐regulation was also positively correlated with infiltrative tumor growth patterns in HCC patients, implying its possible involvement in tumor cell invasiveness 33. Our data directly reveal for the first time to our knowledge that upregulated LMCD1 may intermediate EMT progression and contribute to tumor cell invasiveness and metastasis.

Conversely, IRF5 has been reported as a tumor repressor in breast cancer and negatively regulates CD24 expression, but similarly, no association with date has been shown between IRF5 and EMT to the best of our knowledge 15, 34. We also observed that IRF5 knockdown in preEMT H358 cells increased CD24^+^ cell subpopulation (from 0.6% to 4.1%, Fig. 4D, upper panel). Interestingly, we found that the IRF5‐mediated CD24 repression is particularly significant and important for cells under exposure of EMT‐inducing cytokines such as TGF‐beta. Knockdown of IRF5 led to a much more dramatic increase in CD24^+^ expression in the cells exposed to TGF‐beta/OSM, as compared to untreated cells. Knockdown of IRF5 also significantly increased CD44^+^ expression in postEMT cells, suggesting its critical repressive role in CD44 and CD24 regulation during EMT.

Finally, but no less interestingly, we found that SMAD7, a reported EMT repressor that antagonizes TGF‐beta signaling 35, was actually significantly upregulated in postEMT cells in our EMT model in both H358 and MCF10a cell lines (Table 1). Heikkinen et al. reported that hypoxic conditions could convert SMAD7 function from an inhibitor into a promoter of cell invasion in cancer, which was supported by evidence that increased SMAD7 expression in human cancer correlated with hypoxic gene expression. Further studies are needed to better understand how SMAD7 functions during EMT and metastasis 36.

Taken together, our data help shed new light into the transcriptional landscape of EMT and its regulation. Future studies will need to continue to address how these numerous EMT‐related networks, and regulating genes such as TFs, are dynamically orchestrated during EMT. This could eventually result in development of new prognostic tools and biomarkers, and provide promising targets for the development of novel EMT‐based treatments for cancers.

## Conflict of Interest

None declared.

## Supporting information


**Figure S1.** Top common EMT‐related networks identified by IPA in H358 and MCF10a cells. Network (Nt)1: TGF*β*‐centered pathways. Nt2 involved in NFkB activation. Nt3 closely related to cancer and cellular movement, being involved with WNT pathway and ‘cadherin switch’; Nt 4 related to cellular assembly and organization with vimentin and cytokeratin; Nt5 involved signaling of cellular movement, cell‐to‐cell signaling and interaction.

**Figure S2.** Effects of knockdown of *IRF5* or *LMCD1* on cell morphology in H358 and MCF10 cells that were induced to EMT. Cells were treated with each siRNA for 48 h at 6 nmol/L followed by induction of EMT using TGF‐beta/OSM for 3 days. (A) H358 cells; (B) MCF10a cells. Cells treated with siR‐IRF5 and siR‐LMCD1 showed more EMT morphologic pattern compared with control cells.

**Table S1.** Top 50 genes differentially expressed in H358 and MCF10a cells.
**Table S2.** Common differentially expressed EMT genes shared between H358 and MCF10a.
**Table S3.** 58 core EMT‐related genes shared by our GES and Gupta's GES.
**Table S4.** RT‐qPCR validation of 65 known EMT‐associated genes.
