# Supplementary figures and images for: Transcriptome profiling reveals novel gene expression signatures and regulating transcription factors of TGF β‐induced epithelial‐to‐mesenchymal transition
Source: Cancer Med. 2016 Jun 18;5(8):1962–72. doi: 10.1002/cam4.719 (PMC4971924; doi:10.1002/cam4.719)

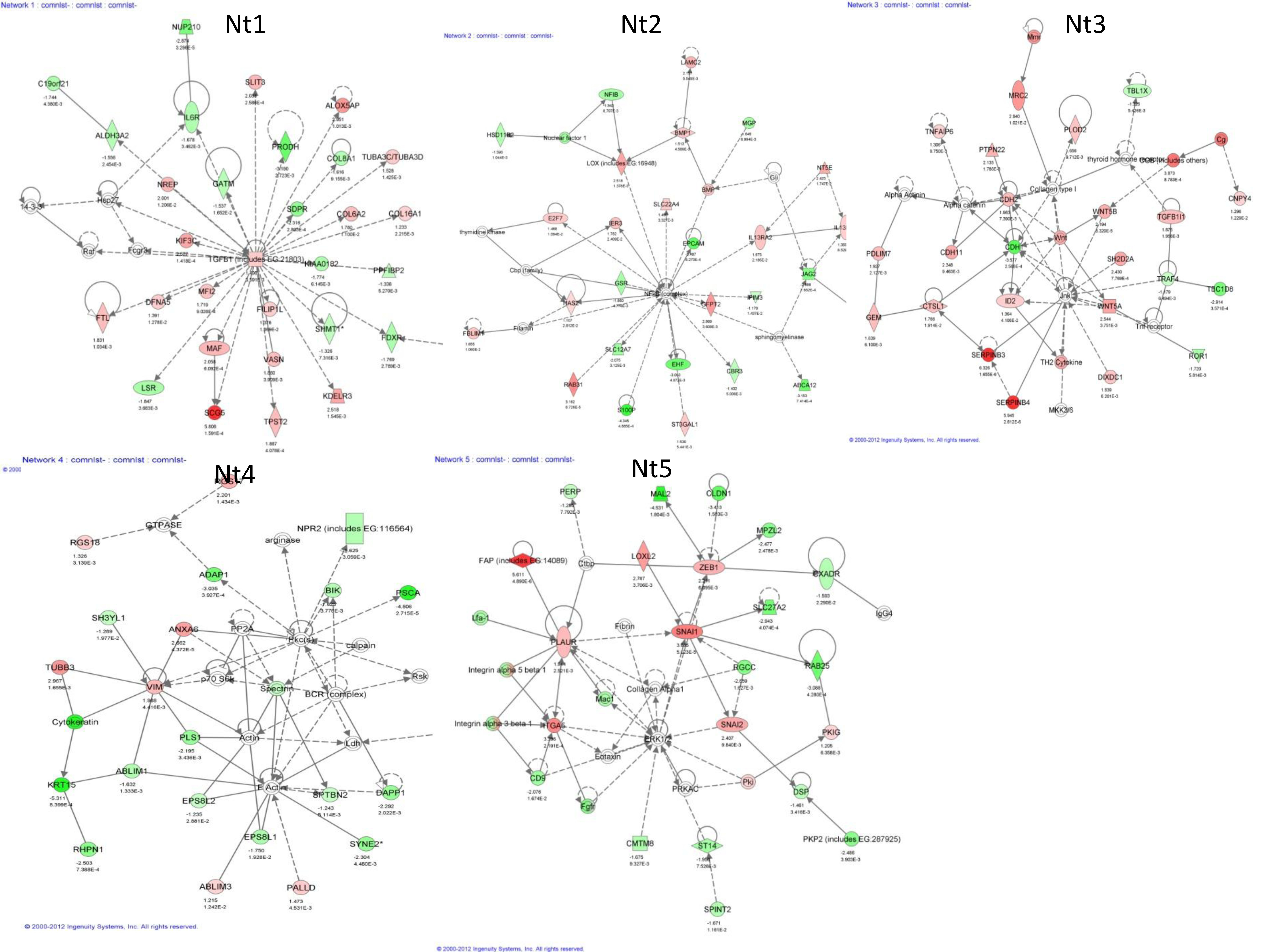

Supplement: Supplementary file 1 — Figure S1. Top common EMT‐related networks identified by IPA in H358 and MCF10a cells. Network (Nt)1: TGFβ‐centered pathways. Nt2 involved in NFkB activation. Nt3 closely related to cancer and cellular movement, being involved with WNT pathway and ‘cadherin switch’; Nt 4 related to cellular assembly and organization with vimentin and cytokeratin; Nt5 involved signaling of cellular movement, cell‐to‐cell signaling and interaction. [file CAM4-5-1962-s001.tif]

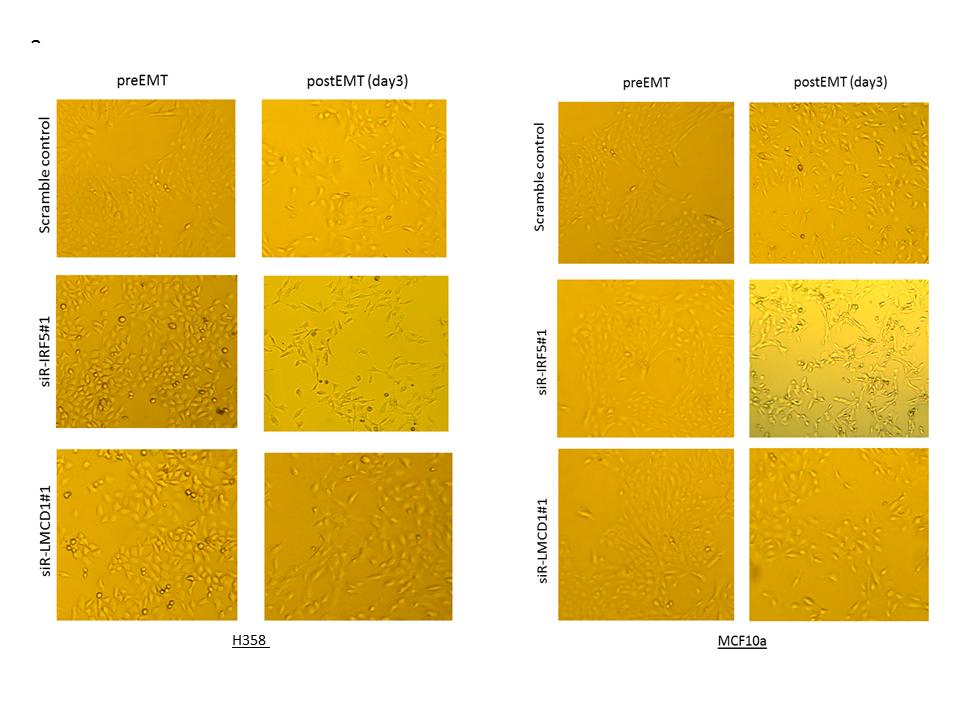

Supplement: Supplementary file 2 — Figure S2. Effects of knockdown of IRF5 or LMCD1 on cell morphology in H358 and MCF10 cells that were induced to EMT. Cells were treated with each siRNA for 48 h at 6 nmol/L followed by induction of EMT using TGF‐beta/OSM for 3 days. (A) H358 cells; (B) MCF10a cells. Cells treated with siR‐IRF5 and siR‐LMCD1 showed more EMT morphologic pattern compared with control cells. [file CAM4-5-1962-s002.tif]
